# Chemical composition and hepatoprotective effect of free phenolic extract from barley during malting process

**DOI:** 10.1038/s41598-018-22808-6

**Published:** 2018-03-13

**Authors:** Meiping Quan, Qin Li, Pei Zhao, Chengrui Tian

**Affiliations:** 1 College of Food Engineering and Nutritional Science, Shaanxi Normal University, Xi’an City, 710062 China; 2 School of Food Science, Shanxi Normal University, Linfen City, 041004 China; 3 The College of Chemistry and Life Science, Weinan Normal University, Wei’nan City, 714000 China

## Abstract

In this study, No.Ganpi4 of barley was steeped and malted to investigate the changes of phenolic compounds during malting process. The free phenolic extract from raw barley (FPEB) was analyzed by HPLC and predominant compounds were (+)-catechin, protocatechuate and quercetin. The FPEB was evaluated for hepatoprotective effect *in vivo* and *in vitro*. Intragastric administration of FPEB (100, 200 and 400 mg/kg/bw) to mice significantly weakened the effects of hepatic damage induced by CCl_4_ toxicity on serum markers, including serum alanine aminotransferase, aspartate aminotransferase, alkaline phosphatase, total-bilirubin, total cholesterol and total triglycerides. FPEB administration also increased the hepatic levels of antioxidant enzymes, such as superoxide dismutase, catalase and glutathione peroxidase. Histopathological examinations further confirmed that FPEB could protect the liver from CCl_4_-induced damage. *In vitro*, the experimental results demonstrated that FPEB could reduce BRL hepatocyte apoptosis and damage induced by CCl_4_. These results suggest that FPEB exerts an effective protection for hepatic injury, and barley has the potential as a functional food to prevent hepatic injury.

## Introduction

The liver plays a critical role in regulating metabolism, clearing xenobiotics and toxins in the body. Hepatic injury associated with liver metabolic dysfunction can result in many disorders like the elevation of hepatic enzymes and emergence of hepatocellular carcinoma^1^. Unfortunately, conventional or synthetic drugs used to treat liver diseases are dissatisfactory because they can exert serious longterm side effects^1–4^. Therefore, it is important to adopt various measures to protect people from hepatic injury^2–4^. Plant polyphenols are one kind of effective and natural bioactive substances, exhibiting different biological activities including antioxidant, antiinflammator activities, and hypoglycaemic and hepatoprotective effects^4–7^. So, a diet rich in plant polyphenols may help to reduce the risk of chronic diseases^7–12^.

Barley (*Hordeum vulgare* L.) is annual herb of the poaceae family and it is the world’s fourth most important cereal crop after wheat, rice, and corn. Barley often served as a key ingredient in the preparation of processed foods like bakery, brewing, and malted products. Furthermore, Barley is increasingly becoming a kind of healthy food due to its high contents of β-glucan, arabinoxylans, bioactive polypeptide, and phenolic compounds^13–15^. Previous studies have revealed that phenolics in barley mainly contains ferulic acid, p-coumaric acid, caffeic acid, protocatechuic acid, flavan-3-ols, and flavonols^16,17^. Phenolics content in cereal vary in different species and different processing methods, and the malting (steeping and germination) process had an effect on the content of barley phenolics^18,19^. Cáceres *et al*.^20^, Ti *et al*.^21^ and Donkor *et al*.^22^ have found that higher phenolic content and stronger antioxidant power could be achieved through germinated grains. Studies revealed that polyphenols were connected with biological activity such as hepatoprotective activity^23–25^. However, to the best of our knowledge, there are few available reports concerning the phenolic compounds and bioactivity of barley. Therefore, the objective of this study was to investigate phenolic content, qualitative and quantitative levels of selected phenolic components of barley during malting process as well as the hepatoprotective effect of FPEB.

## Results and Discussion

### Changes of phenolic content during malting process

**Figure 1:**
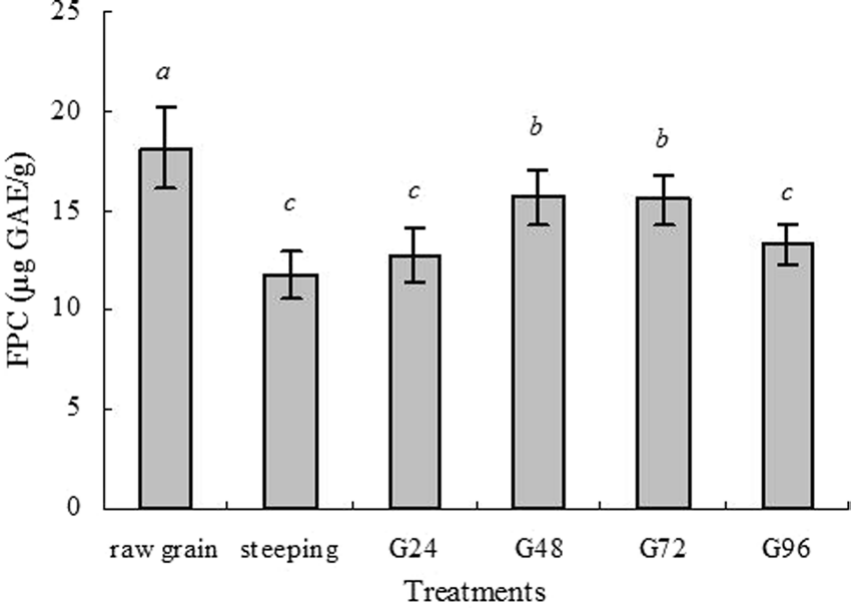
Changes of phenolic content (µg GAE/g DW) during steeping and germination. Values represent means of three independent replicates ± SD. Different letters indicate statistically significant differences between the means (p < 0.05) for free phenolic content.

Free phenolic content (FPC) from barley during malting process were monitored and the results were shown in Fig. 1. FPC in raw barley was 18.18 µg GAE/g DW. Steeping resulted in significant decline in FPC compared with raw grains. A previous study also showed that phenolic content of two barley varieties, Gan4 and Hamelin, significantly decreased after steeping treatment^19^. Another report on sorghum revealed that steeping process resulted in significant decreases of phenolic content^26^, which could be because some phenolics have dissolved into the water during steeping process. Another possible reason was the formation of insoluble complexes between phenolics and proteins^26–28^. During germination, FPC increased progressively with the proceeding of germination. Grain germination can produce various kinds of enzymes to breakdown cell walls surrounding compounds, and total phenols would increase, which was proven by studies on barley^18^ and oat^19^.

### HPLC analysis of free phenolic extract

**Figure 2:**
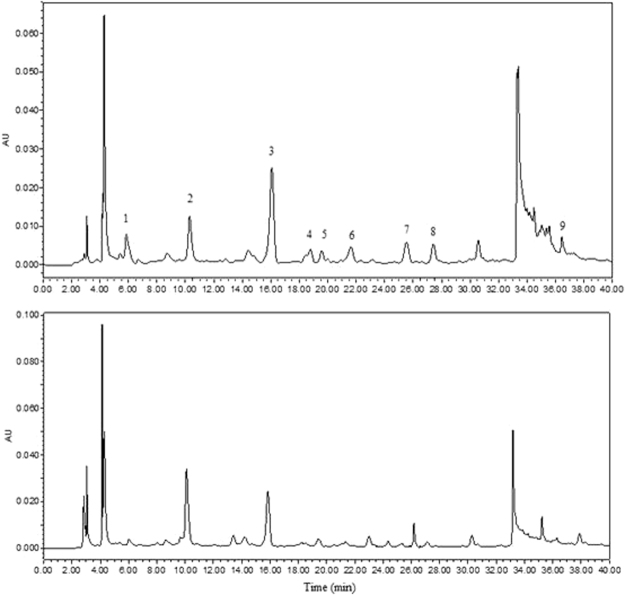
HPLC chromatograms of the free phenolic extracts from barley at wavelength 280 nm. Raw grains (**A**) and sample after 48 h of germination (**B**) are shown. Peaks identification: 1, gallic acid; 2, protocatechuate; 3, (**+**)-catechin; 4, vanillic acid; 5, caffeic acid; 6, (−)-epicatechin; 7, *p*-coumaric acid; 8, ferulic acid; 9, quercetin.

In order to intuitively compare phenolic changes, HPLC chromatogram of extracts for raw grains and sample after 48 h of germination are shown in Fig. 2. It can be seen that nine phenolic compounds such as gallic acid, protocatechuate, (+)-catechin, vanillic acid, caffeic acid, (−)-epicatechin, *p*-coumaric acid, ferulic acid, and quercetin were determined. Meanwhile, although some peaks are strongly response on the UV absorbance at 280 nm, they were not characterized in the present study. For unambiguous identification further studies are required by using authentic compounds. We found there were some new arisen substances while some intrinsic phenolic compounds increased or decreased, and even disappeared after germination. The RP-HPLC quantitative analytical result of the phenolic compounds are shown in Table 1. The data showed that (+)-catechin, protocatechuate, quercetin, ferulic acid and gallic acid were the main phenolic compounds. The (−)-epicatechin, (+)-catechin and *p*-coumaric acid decreased significantly after the steeping stage (p < 0.05). Xu *et al*.^29^ showed a significant decrease of different phenolic acids on oat during steeping; Lu *et al*.^18^ also reported a significant decrease on Gan 4 barley during steeping. Enzymes at germination could stimulate the materials conversion or biosynthesis; meanwhile germination softened the tissue of cell walls and led to easier extraction and better releases of phenolic compounds^30^. In terms of raw grains, almost all monomer phenolic acids were the highest. Steeping caused significant decline of most phenolic acids, especially, p-coumaric acid and caffeic acid contents even declined to lower limit of detection. After steeping, most of the phenolic acids contents reached the highest at G48, and the protocatechuate content at G48 was 2.18 times in comparison with raw grains. Table 1 showed (+)-catechin was the leading phenolic acid; the second was protocatechuate in barley. Lu *et al*.^18^ also concluded (+)-catechin was the most abundant phenolic compound, followed by ferulic acid in the raw barley and corresponding malts.

**Table 1:** Changes of main free phenolic compounds (µg/g DW) during steeping and germination.

	Raw grains	Steeping	G24	G48	G72	G96
gallic acid	1.77 ± 0.07b	1.31 ± 0.05d	1.37 ± 0.06d	1.52 ± 0.04c	1.39 ± 0.06 cd	2.42 ± 0.05a
protocatechuate	2.19 ± 0.03e	2.03 ± 0.03f	2.46 ± 0.05d	4.78 ± 0.05a	4.37 ± 0.02b	2.84 ± 0.04c
vanillic acid	0.70 ± 0.03a	0.40 ± 0.01c	nd	0.49 ± 0.03b	0.40 ± 0.02c	nd
*p*-coumaric acid	0.75 ± 0.04a	nd	nd	0.63 ± 0.01b	0.61 ± 0.01c	0.56 ± 0.02d
ferulic acid	1.19 ± 0.03a	1.08 ± 0.02a	1.09 ± 0.04a	1.18 ± 0.03a	1.21 ± 0.04a	1.11 ± 0.04a
caffeic acid	0.86 ± 0.04a	nd	nd	nd	nd	nd
(+)-catechin	6.64 ± 0.02a	4.50 ± 0.07d	5.37 ± 0.04b	4.36 ± 0.03e	5.20 ± 0.04c	4.23 ± 0.02e
(−)-epicatechin	2.07 ± 0.02a	0.86 ± 0.03f	0.94 ± 0.09d	1.11 ± 0.04b	0.97 ± 0.02c	0.88 ± 0.03e
quercetin	2.02 ± 0.05a	1.60 ± 0.05b	1.52 ± 0.04c	1.62 ± 0.05b	1.43 ± 0.02d	1.31 ± 0.02e

Values represent means of three independent replicates ± SD. Different letters within a column indicate statistically significant differences between the means (p < 0.05). nd, not detect.

### Hepatoprotective effects of FPEB *in vivo*

#### Effect of FPEB on liver indices

The effects of the FPEB on the liver indices are shown in Table 2. Compared with the control group, the mice in the CCl_4_-treated group had a significant increase in liver index (p < 0.01), indicating that their hepatic tissue have been severely damaged, and that the livers were swollen as a result of exposure to CCl_4_ toxicity, which was supported by previous study^24^. However, the group treated with three doses of FPEB for 3 weeks showed a significantly lower (p < 0.05) liver index compared with that of the CCl_4_-treated group, which revealed that the administering of the tested FPEB had a preventive effect against CCl_4_-induced hepatic damage in the mice.

**Table 2:** Effects of FPEB on the liver indexes in CCl_4_ intoxicated mice.

Groups	Liver index	Liver weight (mg)	Body weight (g)
Control	44.68 ± 1.02b	1220.2	27.3
1% CCl_4_ treatment (5 mL/kg b.w.)	50.57 ± 2.65a	1335.1	26.4
FPEB (100 mg/kg) + CCl_4_	46.01 ± 2.19b	1270.4	27.6
FPEB (200 mg/kg) + CCl_4_	45.20 ± 1.95 b	1151.5	25.5
FPEB (400 mg/kg) + CCl_4_	45.31 ± 1.82b	1183.1	26.1
BP (200 mg/kg) + CCl_4_	46.28 ± 2.05b	1228.2	26.6

Values represent means of three independent replicates ± SD. Different letters in the same column indicate statistically significant differences among the different groups (p < 0.05).

#### Effects of the FPEB on serum AST, ALT, TC, TG, AKP and albumin activity levels

Hepatic function is often measured by serum ALT and AST enzymatic activities, and there are still other important indexes, such as TC, TG, AKP and albumin level in serum to assess hepatic function. As depicted in Table 3, the serum AST and ALT enzymatic activities of the CCl_4_-treated group sharply increased from 28 to 109.0 U/L (p < 0.05) and from 28.0 to 114.3 U/L (p < 0.05) compared with the control group, respectively. Treatments with FPEB at different dosage reduced the AST and ALT activities (p < 0.05). The results exhibited dose-effect relationship, and high-dosage (400 mg/kg/bw) showed particularly better effect, which is even close to the BP group compared with those of the CCl_4_-treated group.

**Table 3:** Effects of FPEB on the serum levels of AST, ALT, TC, TG, AKP and Albumin.

Groups	AST (U/L)	ALT (U/L)	TC (mmol/L)	TG (mmol/L)	AKP (U/L)	Albumin (g/L)
Control	28.0 ± 3.5e	20.8 ± 2.3e	3.6 ± 0.5c	1.1 ± 0.15c	11.8 ± 1.7c	36.7 ± 3.5a
1% CCl_4_ treatment	109.0 ± 7.2a	114.3 ± 5.8a	5.4 ± 0.8a	1.7 ± 0.25a	20.9 ± 2.7a	23.6 ± 2.8c
FPEB (100 mg/kg) + CCl_4_	98.6 ± 4.8b	93.6 ± 4.9b	4.6 ± 0.6ab	1.4 ± 0.2b	18.9 ± 3.9ab	25.8 ± 3.8bc
FPEB (200 mg/kg) + CCl_4_	78.3 ± 4.8c	82.9 ± 3.6c	4.1 ± 0.5bc	1.2 ± 0.1bc	15.4 ± 2.5bc	28.1 ± 4.1bc
FPEB (400 mg/kg) + CCl_4_	65.1 ± 4.7d	72.0 ± 3.8d	3.9 ± 0.9bc	1.2 ± 0.1bc	15.9 ± 2.2bc	30.6 ± 4.3ab
BP (200 mg/kg) + CCl_4_	68.1 ± 5.0d	73.8 ± 5.2d	3.9 ± 0.9bc	1.0 ± 0.2c	13.9 ± 1.2c	35.3 ± 2.9a

Values represent means of three independent replicates ± SD. Different letters within a column indicate statistically significant differences between the means (p < 0.05).

Results of serum TC and TG levels are also shown in Table 3. The serum TC and TG level was significantly increased in the CCl_4_-treated group compared with the normal control group (p < 0.05). FPEB pretreatment at a dose of 100, 200 and 400 mg/kg prior to CCl_4_ injection markedly prevented the elevation (p < 0.05) of TG. However, improvement effect of FPEB at low-dosage (100 mg/kg/bw) on TC level was not obvious, only slightly lower compared with the CCl_4_-treated group. Nonetheless, FPEB pretreatment at 200 and 400 mg/kg/bw relatively had a good protective effect on the serum TC and TG levels, similar to that of the reference compound BP. As shown in Table 3, in the control group, the serum AKP and albumin levels were 11.8 U/L and 36.7 g/L, respectively. After CCl_4_ exposure, serum AKP markedly increased by 43.3% while albumin levels decreased by 35.5% (p < 0.05). However, the CCl_4_-induced elevation of these values was significantly weakened by pretreatment with FPEB at 200 and 400 mg/kg/bw. Pretreatment with FPEB at a low dose of 100 mg/kg/bw led to a slight change in AKP and albumin levels, yet, it was insignificantly different from that of the CCl_4_-treated group (p > 0.05).

#### Effects of FPEB on SOD, CAT, GPx and lipid peroxidation

Antioxidant enzymes, such as GPx (glutathione peroxidase), CAT (catalase) and SOD (superoxidedismutase) play an important role in effectively suppressing free radical oxidation damage in human metabolism. Furthermore, they are also the major elements in human bodies that increase immunity, prevent diseases and balance the metabolism process of oxygen free radical and lipid peroxidation reaction. Therefore, the activities of these antioxidant enzymes are closely related to liver detoxification function^24^. As shown in Table 4, the levels of hepatic SOD, CAT, and GPx activities in the CCl_4_-treated group were significantly lower than those of the control group (p < 0.01). In the CCl_4_-treated group, the activity levels of the hepatic antioxidant enzymes SOD, CAT and GPx decreased respectively by 46.7%, 35.4% and 35.4% as compared to the control group. On the contrary, the groups treated with FPEB at three different doses significantly increased (p < 0.05) in levels of SOD, CAT and GPx activities, and the preventive effect of FPEB treatment at 400 mg/kg/bw was similar to that of BP treatment. In this study, lipid peroxidation was measured and expressed as nmol of malondialdehyde equivalents (MDAeq) per milligram of proteins according to kit instruction. The effects of FPEB pretreatment on the CCl_4_-induced alteration of MDA level are presented in Table 4. The liver lipid peroxidation level or MDA content was significantly increased in the CCl_4_-treated group compared with that of the control group (p < 0.05). However, the FPEB treatment at 400 mg/kg/bw markedly relieved the induced elevation in the lipid peroxidation level (p < 0.05), and exhibited a positive protective effect which similar to that of BP treatment.

**Table 4:** Protective effects of FPEB on the hepatic SOD, CAT, GPx and lipid peroxidation.

Groups	SOD (U/mg prot)	CAT(U/mg prot)	GPx (μmol/g prot)	MDA (nmol/mg prot)
Control	52.21 ± 2.98a	16.52 ± 1.69a	250.98 ± 12.58a	3.25 ± 0.88c
1% CCl_4_ treatment	27.82 ± 3.77e	10.68 ± 1.42c	162.19 ± 10.31d	7.50 ± 1.31a
FPEB (100 mg/kg) + CCl_4_	35.32 ± 2.69d	12.31 ± 0.85bc	197.70 ± 15.07c	6.77 ± 0.69a
FPEB (200 mg/kg) + CCl_4_	38.71 ± 3.03cd	14.42 ± 1.52ab	209.78 ± 10.38bc	5.81 ± 1.52ab
FPEB (400 mg/kg) + CCl_4_	42.28 ± 3.79bc	13.92 ± 1.74ab	220.68 ± 16.06bc	4.49 ± 1.06bc
BP (200 mg/kg) + CCl_4_	45.54 ± 2.82b	14.59 ± 1.26ab	230.33 ± 17.68ab	3.86 ± 1.03bc

Values represent means of three independent replicates ± SD. Different letters within a column indicate statistically significant differences (p < 0.05).

#### Histopathological examination of mouse livers

**Figure 3:**
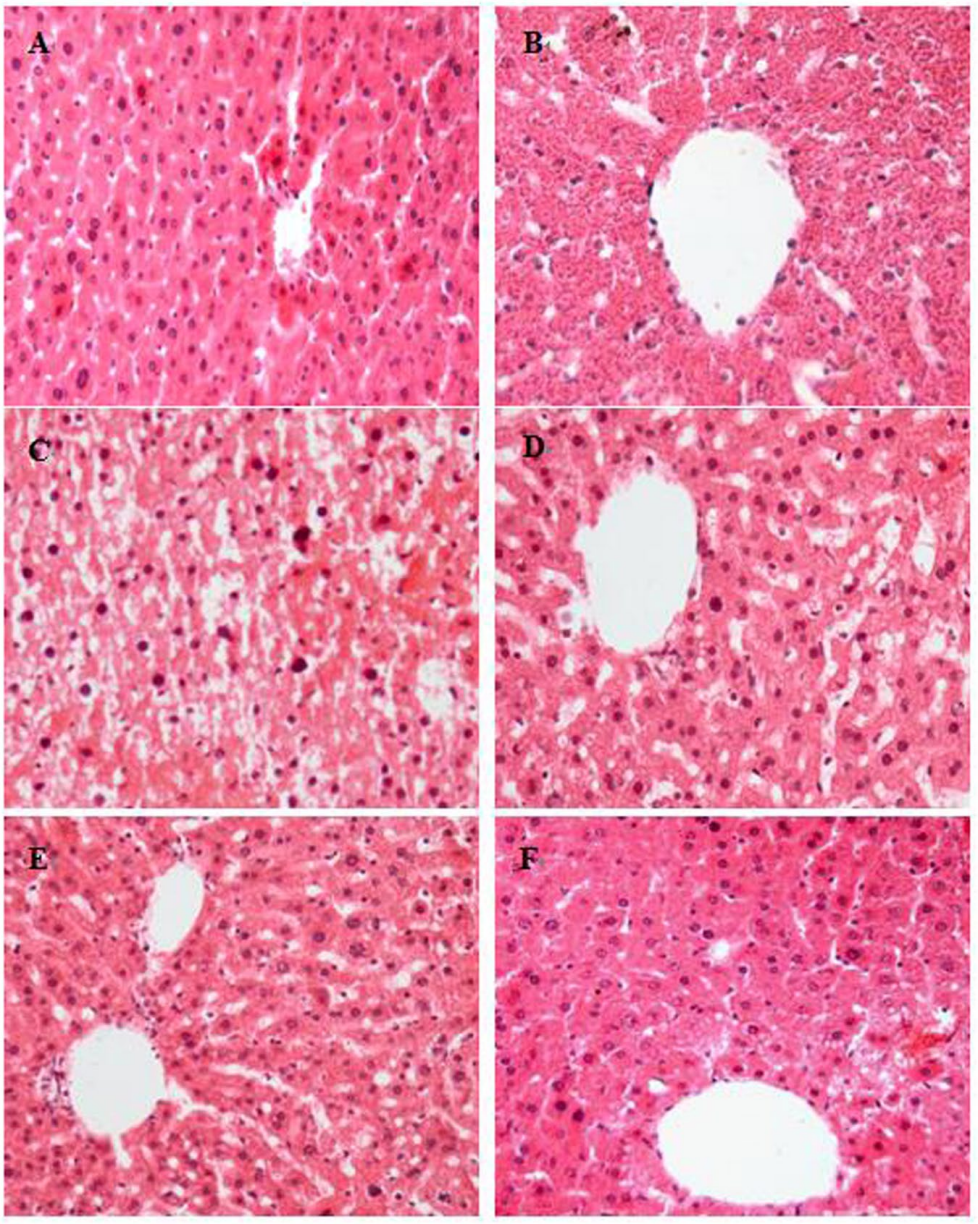
Preventive effects of FPEB against CCl_4_-induced liver histopathological changes in mice (original magnification of ×400; hematoxylin and eosin). (**A**) Control group, (**B**) CCl_4_-intoxicated control group, (**C**) 100 mg/kg/bw FPEB (low-dose group)+CCl_4_, (**D**) 200 mg/kg/bw FPEB (medium-dose group)+CCl_4_, (**E**) 400 mg/kg/bw FPEB (high-dose group)+CCl_4_, (**F**) BP positive control group (200 mg/kg/bw)+CCl_4_.

Histopathological examination can provide firsthand evidence of the effects of the investigated components against acute CCl_4_-induced hepatic injury^7^. As shown in Fig. 3, hepatic tissue of the control group contained typical, regular and intact hepatic cellular structure, along with a well-preserved cytoplasm, a prominent nucleus and nucleolus and visible central veins (Fig. 3A). In contrast, hepatic tissue of CCl_4_ treatment exhibited severe histological changes and damage with vague cellular boundary, extensively deformed hepatocyte and collapse of parenchyma (Fig. 3B). However, the hepatic histopathological changes induced by CCl_4_ exposure were gradually ameliorated and recovered by FPEB treatment with the increase of dose (Fig. 3D,E). The FPEB administered at 400 mg/kg/bw was obviously effective and even close to the positive control BP drug in hepatic protection effect. This is because the hepatic cells of high-dose and positive group had a comparatively regular appearance with a well-preserved cytoplasm and prominent nuclei (Fig. 3E,F), which showed that FPEB exerted a good hepatoprotective effect. The histopathological result also supported our above biochemical results regarding the serum hepatotoxic markers and hepatic oxidative stress systems.

#### Hepatoprotective effects of FPEB *in vitro*

The results of CCl_4_-induced injury and cell viability of BRL hepatocytes *in vitro* are presented in Table 5 according to Gao *et al*.^24^. Cell viability significantly decreased by 74.0% in the CCl_4_-treated group compared with the control group. There was a significant increase of cell viability in the FPEB-treated groups compared with that of the CCl_4_-treated group (p < 0.01), and also there were obvious dose–response way between cell viability and the tested concentration of FPEB. However, cell viability of FPEB-treated groups was far lower than that of control group. The LDH, ALT and AST activities in CCl_4_-treated BRL hepatocytes supernatants with the administration of FPEB were also evaluated. Similarly, ALT and AST are important indicators of liver injury as previously mentioned^31^. As shown in Table 5, the AST, ALT and LDH activities were significantly higher in the supernatants of the CCl_4_-treated group compared with those of the control group (p < 0.01) and the LDH, AST and ALT levels was increased by 168.8%, 330.7% and 230.6% respectively. However, pretreatment with FPEB at 25, 50 and 100 μg/mL reduced the elevation of the AST, ALT and LDH activities caused by CCl_4_ treatment (p < 0.01) in a dose-dependent manner. No difference in cell viability, LDH, ALT and AST activities was found between BP and treatment group with 100 μg/mL FPEB. This strongly proved and explained the high cell viability in FPEB-treated groups. The results indicate that FPEB could reduce BRL hepatocyte apoptosis and damage caused by CCl_4_ exposure *in vitro*.

**Table 5:** Effects of pretreatment with FPEB on cell viability and LDH, AST and ALT activities of BRL hepatocyte_._

Groups	Cell viability (%)	LDH (U/L)	AST (U/L)	ALT (U/L)
Control	94.11 ± 4.39a	42.96 ± 3.23d	10.29 ± 1.68e	0.98 ± 0.12d
CCl_4_ alone	24.51 ± 2.79d	115.49 ± 8.37a	44.32 ± 1.70a	3.24 ± 0.12a
FPEB (25 μg/mL) + CCl_4_	45.74 ± 2.87c	100.28 ± 9.13ab	33.77 ± 1.72b	3.08 ± 0.13a
FPEB (50 μg/mL) + CCl_4_	67.33 ± 3.05b	86.69 ± 6.10b	26.44 ± 1.63c	2.76 ± 0.09b
FPEB (100 μg/mL) + CCl_4_	74.51 ± 4.08b	64.40 ± 2.72c	18.64 ± 0.56d	2.27 ± 0.06c
BP (50 μg/mL) + CCl_4_	73.62 ± 3.14b	65.56 ± 1.78c	17.85 ± 0.86d	2.41 ± 0.18c

Values represent means of three independent replicates ± SD. Different letters within a column indicate statistically significant differences between the means (p < 0.01).

It is well-known that phenolic compounds and their content in the plants are mainly responsible for the antioxidant potential of plants^32^. Meanwhile, medicinal and beneficial functions of plants are also closely related with their natural antioxidants present in plants. Previous studies have proven that phenolic extracts from some plants exhibited certain biological activities including DNA damage protective activity, inhibition of lipid peroxidation and hepatoprotective effect induced by CCl_4_ exposure^23,24,33^. Accordingly in this study, a relatively higher phenolic compound existed in FPEB could explains the positive or protective ability of FPEB against the hepatic injury caused by CCl_4_ exposure in mice and BRL hepatocytes. However, after being ingested, the metabolism of polyphenol compounds resulted in the formation of new and often completely different compounds compared to the ingested molecules in the body^34^. Therefore, the hepatoprotective effect of free phenolic extract from barley still needs to be further studied by *in vivo* experiments.

## Conclusion

In conclusion, the data obtained from this study indicates that steeping and malting resulted in significant decreases in FPC compared to raw grains. The (+)-catechin, protocatechuate, quercetin, ferulic acid and gallic acid were the main phenolic compounds in barley. Additionally, the result of the animals experiment indicates that pretreatment with FPEB was effective in the prevention of oxidative stress and liver damage induced by CCl_4_ in rats, as revealed by the marked decrease in the hepatic lipid peroxidation content, the reduced serum AST, ALT, TC, TG and AKP levels, the enhanced hepatic SOD, CAT and GPx activities. Furthermore, histopathological morphologic improvement also demonstrates the beneficial effects of FPEB pretreatment. The results *in vitro* experiment also indicates that FPEB could decrease BRL hepatocyte apoptosis and damage induced by CCl_4_. Therefore, the phenolic extract from barley could be used as a hepatoprotective agent. This is the first report on the hepatoprotective effect of phenolics from barley. These results are expected to provide a scientific basis for the further development and exploitation of barley resource.

### Materials and methods

Standards of phenolic acid, including gallic acid, (+)-catechin, protocatechuate, (−)-epicatechin, caffeic acid, vanillic acid, p-coumaric acid and ferulic acid were obtained from Sigma Chemical Co. (St. Louis, MO, USA), Carbon tetrachloride (CCl_4_), acetone, ethyl acetate and sodium hypochlorite from the Tianjin Chemical Reagent Co. (Tianjin, China), and bifendate pills (BP) were obtained from Beijing Union Pharm (Beijing, China). Haematoxylin and eosin (H&E) were obtained from the Shanghai jianglai biological technology Co. Ltd. (Shanghai, China). The diagnostic kits for liver and serum index were obtained from the Jiancheng Institute of Biotechnology (Nanjing, China), and the BRL hepatocytes were purchased from the Institutes for Biological Sciences Cell Resource Center (Shanghai, China). All of the other chemicals and reagents used in the experiments were of analytical grade.

### Preparation of barley samples

Barley cultivar (*Hordeum uulgure*) Ganpi4, a naked variety, was obtained from Lanzhou in Gansu province during the crop year. Barley grains were selected in whole grain, sterilized using a 0.05% solution of sodium hypochlorite for 30 s, and then washed three times with deionized water before steeping. The grains were spread out on clean and humid gauze in thermostat incubator (HWS-280, Hangzhou Huier Instruments, Zhejiang, China) at 16 °C for 12 hours. The grains were marked as steeping sample. Other barley grains was continued to sprout for an established time, and the samples were respectively taken out from incubator at 24 h, 48 h, 72 h and 96 h, which were successively marked as G24, G48, G72 and G96. After the treatment mentioned above, samples were oven-dried at 35 °C temperature, and then milled with a micro plant grinding machine (HuaChen1000; Zhejiang, China) to a powder about 0.5 mm. Samples with different treatment were stored at −40 °C prior to analysis. Raw grains were also freeze-dried and used as reference samples in all performed analyses.

### Extraction of free phenolic compounds

Free phenolic compounds were extracted according to the method with minor modifications^35^. Briefly, 5 g milled samples was defatted with 50 mL of hexane at room temperature by a rotary shaker (HY-5, Jintan billion electronics co., Ltd., Jiangsu, China.) and then treated with 25 mL of methanol for 20 min in the rotary shaker. The homogenates were centrifuged (Eppendorf5810R, Germany) at 4000 g for 10 min. The supernatant was removed following centrifugation while extraction of the precipitate was repeated twice. The three supernatants were collected, combined, and evaporated to dryness at 45 °C under vacuum and then reconstituted to a final volume of 10 mL with methanol. The extracts were then stored at −20 °C until analyzed.

### Analytical methods

#### Determination of phenolic content

Phenolic content was determined in terms of Folin-Ciocalteu colorimetric method described by Payet *et al*.^36^ with minor modifications. The 0.5 mL diluted extracts or standard solutions was mixed with 2.5 mL deionized water and 0.5 mL 1.0 M Folin-Ciocalteu reagent in volumetric flask. After 10 minuets, 1.5 mL 7.5% sodium carbonate was added and mixed thoroughly. After two hours of incubation at room temperature, the absorbance at 765 nm was detected with a UV-2000 spectrometer (Hitachi). Methanol was used as the blank and gallic acid (GA) was used for calibration of standard curve (0–10 μg/mL). The total phenolic content was expressed as micrograms of gallic acid equivalents (GAE) per gram of dry weight (µg of GAE/g of dw).

#### HPLC Analysis of free phenolic extract from barley during malting process

HPLC analyses were performed using a Waters 1525 pump (Waters, Milford, MA) equipped with Waters 2487 Diode Array Detector (DAD) (Waters, Milford, MA) and a reversed-phase C18 column (Alltech, Allsphere ODS-2, 5 μm, 150 mm × 4.6 mm). Elution was carried out with containing solvent A (0.1% methanoic acid in high purity water) and solvent B (0.1% methanoic acid in acetonitrile) as a mobile phase. The specific gradient procedure was as follows: 0–25 min, B from 7% to 18%; 25–30 min, B from 18% to 22%; 30–31 min, B from 22% to 50%; 31–40 min, and 50% of solvent B. The solvent flow rate was 1.0 mL/min at room temperature, and the injection volume was 20 µL. The duration of a single run was 40 min while the monitored wavelength was 280 nm. The phenolic extracts and standard compounds were analyzed under the same analysis conditions, and all of the above experiments were replicated three times. Identification of the main phenolic acids was performed by comparisons to the retention time and UV spectra of authentic standards. The concentrations of phenolic compounds in barley extracts were calculated according to standard curves, and the results for the main phenolic compounds were expressed in micrograms per gram of dry weight (µg/g of dw).

### Determination of the hepatoprotective effect of FPEB ***in vivo***

#### Animals and experimental design

Sixty Kunming male mice (weight 20 ± 2 g) were obtained from the Experimental Animal Center of Fourth Military Medical University, and the study was approved by the Medical Ethics Committee of the University. They were allowed free access to tap water and rodent chow, housed under the standard conditions with 12/12 h light-dark cycle at a temperature of 22 ± 2 °C, and a humidity of 60 ± 5%. The experimental animal procedures were in accordance with the Regulations of Experimental Animal Administration of the Fourth Military Medical University Committee on Animal Care and Use (approval number, SYXK-007-2007).

After three days of environmental adaptation, the mice were randomly divided into the normal control, CCl_4_-intoxicated, the positive bifendate (BP) group and three experimental groups. The three test groups were respectively given 100, 200, or 400 mg/kg/bw of FPEB (0.3 mL, ig) once daily for 21 consecutive days. The animals from the normal and CCl4-intoxicated groups were also given the same volume of physiological saline, and the positive bifendate (BP) group received 200 mg/kg/bw reference drug BP (0.3 mL, ig) once daily for 21 consecutive days. On the 22nd day, all the groups except control group were given a 1% CCl_4_/peanut oil dissolved in peanut oil (v/v) by intraperitoneal injection (ip, 0.3 mL), while the control group mice merely received peanut oil. After two hours, all the groups were fasted, but allowed water and libitum as usual, for 24 h. The mice were then sacrificed for obtaining blood and livers. Serum was separated by centrifugation at 3000 rpm for 10 min, and then stored at −20 °C until analysis. A liver was dissected out from each mouse and washed immediately with ice cold saline to remove as much blood as possible, and then stored at −40 °C until further analysis. The liver indexes were calculated based on records of body weight, and the corresponding liver weights of every mouse (liver index = liver weight/body weight × 100%).

#### Determination of Biochemical Parameters in Serum-AST, ALT, TC, TG, AKP and albumin

Liver damage was assessed by estimating the enzymatic activities of serum AST, ALT and AKP, as well as serum TC, TG and albumin level, using the corresponding commercial kits (Nanjing Jiancheng Institute of Biotechnology, China), respectively. The results were expressed according to the kit instruction.

#### Determination of antioxidant enzyme activities

Liver homogenate 10.0% (w/v) was prepared with frozen normal saline and centrifuged at 3000 rpm for 15 min. The homogenate supernatant was used for the measurement of SOD, CAT and GPx. All of these enzymes were determined according to the instructions on the commercial kits (Nanjing Jiancheng Institute of Biotechnology, China). SOD and CAT were expressed as units/mg protein (U/mgprot) and GPx was expressed as μmol/gprot.

#### Determination of lipid peroxidation and parameters of hepatic function in Liver

Lipid peroxidation was measured according to MDA diagnostic kits (Nanjing Jiancheng Institute of Biotechnology, China). The results were expressed as nmol/mg protein. The Parameters (TC and TG) of hepatic function was measured using commercially available diagnostic kits, and the results were expressed as mmol/gprot.

#### Histopathological assessment of liver damage

Samples of liver tissues were fixed in 10% neutral buffered formalin for 24 h. Specimens were dehydrated with ethanol solution of different concentration, and embedded in paraffin; these specimens were also sectioned at 5 μm and stained with hematoxylin–eosin (H&E) for histopathological examination. The histopathological changes in the sections were observed by a light photomicroscope. Finally, the images were examined and evaluated for pathological change analysis

### Determination of the hepatoprotective effect of FPEB ***in vitro***

#### Determination of cell viability

BRL hepatocytes were seeded at 2.5 × 10^4^/mL, and 100 μL was added in each 96-well plate. After 12 h of incubation, 100 μL of different concentrations of FPEB (100, 50, 25 μg/mL, in DMEM containing 0.1% dimethyl sulphoxide) was added. The CC1_4_-treated group and the control group were given 100 μL of DMEM. Then, after 12 h of incubation, 50 μL PBS was added to the control group, while the other groups were given 50 μL CC1_4_ (100 mM) to induce cell injury. 6 h later, cell viability was determined. Cell viability was assessed by microculture tetrazolium assay and the results were expressed as percent cell viability.

#### Determination of the LDH, AST and ALT activities in supernatants

BRL hepatocytes were seeded at 2.5 × 10^4^/mL, and 500 μL was added in each 24-well plate. Following 12 h of incubation, 500 μL of different concentrations of FPEB (100, 50, 25 μg/mL, in DMEM containing 0.1% dimethyl sulphoxide) was added. The CC1_4_-treated group and the control group were given 500 μL of DMEM. After 12 h of incubation, 250 μL PBS was added to the control group, while the other groups were given 250 μL CC1_4_ (100 mM) to induce cell injury. The LDH, AST and ALT activities in supernatants were determined after 6 h.

#### Statistical analysis

The experimental results were expressed as the mean ± standard deviation (SD). The data obtained were analysed using one-way analysis of variance (ANOVA) and Duncan’s multiple range tests. Difference was considered significant at the level p < 0.05 or p < 0.01.

## References

[1] Ishibashi H, Nakamura M, Komori A, Migita K, Shimoda S (2009). Liver architecture, cell function, and disease. Semin. Immunopathol..

[2] Bhoopat L (2009). Hepatoprotective effects of lychee (*Litchi chinensis Sonn*.): A combination of antioxidant and antiapoptotic activities. J. Ethnopharmacol..

[3] Samojlik I, Lakic N, Mimica N, Dakovic K, Bozin B (2010). Antioxidant and hepatoprotective potential of essential oils of coriander (*Coriandrum sativum L*.) and caraway (*Carum carvi L*.) (Apiaceae). J. Agric. Food Chem..

[4] Shimoda H (2008). Walnut polyphenols prevent liver damage induced by carbon tetrachloride and D-galactosamine: Hepatoprotective hydrolyzable tannins in the kernel pellicles of walnut. J. Agric. Food Chem..

[5] Giacometti J, Muhvić D, Pavletić A, Ðudarić L (2016). Cocoa polyphenols exhibit antioxidant, antiinflammatory, anticancerogenic, and antinecrotic activity in carbon tetrachlorideintoxicated mice. J. Funct. Foods..

[6] Man S (2016). Chemical composition and hypoglycaemic effect of polyphenol extracts from Litchi chinensis seeds. J. Funct. Foods..

[7] Ma T (2015). Chemical composition and hepatoprotective effects of polyphenols extracted from the stems and leaves of *Sphallerocarpus gracilis*. J. Funct. Foods.

[8] Kazuo Y, Motoki T, Yukio Y (2015). Dietary polyphenols regulate endothelial function and prevent cardiovascular disease. Nutrition.

[9] Scalbert A, Manach C, Morand C, Rémésy C, Jiménez L (2005). Dietary polyphenols and the prevention of diseases. Crit. Rev. Food Sci..

[10] Madhujith T, Shahidi F (2007). Antioxidative and antiproliferative properties of select barley (*Hordeum vulgare* L.) cultivars and their potential for inhibition of low density lipoprotein (LDL) cholesterol oxidation. J. Agric. Food Chem..

[11] Maillard MN, Soum MH, Boivin P (1996). Antioxidant activity of barley and malt: relationship with phenolic content. Lebensm-wiss Technol..

[12] Yang QM, Pan XH, Kong WB (2010). Antioxidant activities of malt extract from barley (*Hordeum vulgare* L.) toward various oxidative stress *in vitro* and *in vivo*. Food Chem..

[13] Alu’datt M (2012). Effects of barley flour and barley protein isolate on chemical, functional, nutritional and biological properties of Pita bread. Food Hydrocolloids.

[14] Madhujith T, Izydorczyk M, Shahidi F (2006). Antioxidant properties of pearled barley fractions. J. Agric. Food Chem..

[15] Sharma P, Gujral HS (2010). Antioxidant and polyphenol oxidase activity of germinated barley and its milling fractions. Food Chem..

[16] Goupy P, Hugues M, Boivin P (1999). Antioxidant composition and activity of barley (*Hordeum vulgare*) and malt extracts and of isolated phenolic compounds. J. Sci. Food Agr..

[17] Hernanz D (2001). Hydroxycinnamic acids and ferulic acid dehydrodimers in barley and processed barley. J. Agric. Food Chem..

[18] Lu J, Zhao HF, Chen J (2007). Evolution of phenolic compounds and antioxidant activity during malting. J. Agric. Food Chem..

[19] Kaukovirta-Norja A, Wilhelmsson A, Poutanen K (2004). Germination: a means to improve the functionality of oats. Agric. Food Sci..

[20] Cáceres PJ, Martínez-Villaluenga C, Amigo L, Fris J (2014). Maximising the phytochemical content and antioxidant activity of Ecuadorian brown rice sprouts through optimal germination conditions. Food Chem..

[21] Ti H, Zhang R, Zhang M, Li Q, Wei Z (2014). Dynamic changes in the free and bound phenolic compounds and antioxidant activity of brown rice at different germination stages. Food Chem..

[22] Donkor O, Stojanovska L, Ginn P, Ashton J, Vasiljevic T (2012). Germinated grains-sources of bioactive compounds. Food Chem..

[23] Chen J (2012). Studies of the protective effect and antioxidant mechanism of blueberry anthocyanins in a CC1_4_-induced liver injury model in mice. Food Agr. Immunol..

[24] Gao CY, Tian CR, Zhou R, Zhang RG, Lu YH (2014). Phenolic composition, DNA damage protective activity and hepatoprotective effect of free phenolic extract from *Sphallerocarpus gracilis* seeds. Int. Immunopharmacol..

[25] Tian LM (2012). Chemical composition and hepatoprotective effects of polyphenol rich extract from *Houttuynia cordata* Tea. J. Agric. Food Chem..

[26] Dicko MH, Gruppen H, Traore AS, Van-Berkel WJH, Voragen AGJ (2005). Evaluation of the effect of germination on phenolic compounds and antioxidant Activities in sorghum varieties. J. Agric. Food Chem..

[27] Beta T, Rooney LW, Marovatsanga LT, Taylor JR (1999). N, Phenolic compounds and kernel characteristics of Zimbabwean sorghums. J. Agric. Food Chem..

[28] Adom KK, Liu RH (2002). Antioxidant activity of grains. J. Agric. Food Chem..

[29] Xu JG, Tian CR, Hu QP, Luo JY, Wang XD (2009). Dynamic changes in phenolic compounds and antioxidant activity in oats (*Avena nuda* L.) during steeping and germination. J. Agric. Food Chem..

[30] Woffenden HM, Ames JM, Chandra S, Anese M, Nicoli MC (2002). Effect of kilning on the antioxidant and pro-oxidant activities of pale malts. J. Agric. Food Chem..

[31] Yang X, Dong C, Ren G (2011). Effect of soyasaponins-rich extract from soybean on acute alcohol-induced hepatotoxicity in mice. J. Agric. Food Chem..

[32] Ozsoy N, Can A, Yanardag R (2008). Antioxidant activity of *Smilax excelsa* L. leaf extracts. Food Chem..

[33] Gao CY (2011). Main nutrients, phenolics, antioxidant activity, DNA damage protective effect and microstructure of *Sphallerocarpus gracilis* root at different harvest time. Food Chem..

[34] Del Rio D (2013). Dietary (poly) phenolics in human health: structures, bioavailability, and evidence of protective effects against chronic diseases. Antioxid. Redox. Signal..

[35] Ti H, Li Q, Zhang R, Deng Y, Wei Z (2014). Free and bound phenolic profiles and antioxidant activity of milled fractions of different indica rice varieties cultivated in southern China. Food Chem..

[36] Payet B, Cheong AS, Smadja J (2006). Comparison of the concentrations of phenolic constituents in cane sugar manufacturing products with their antioxidant activities. J. Agric. Food Chem..

